# One step forward in health promotion

**Published:** 2012-09-25

**Authors:** M Rosu

**Affiliations:** Romanian-American University, Bucharest, Romania

**Keywords:** Thalassemia, Amniocentesis, CVS, Exjade, chelator

## Abstract

Thalassemias are inherited blood disorders. In 2011, there was no clear situation regarding the number of thalassemia or other hemoglobinopathy patients in Romania. The luck of information has led to an increased number of patients registered by Fundeni Hospital, Bucharest in the last years. The main goal of this article is to underline the importance of implementing a screening program in Romania for thalassemia and other hemoglobinopathies, parallel with an awareness program. The importance of this objective is strongly supported by the following example: in Sardinia, thalassemia major was present in one out of 250 births, and has declined to one out of 4000 births after they implemented the screening program. The article is written by Monica Rosu, a participant to the World Wide Opportunities for Woman course, held in Canada, in 1999 and states one of the first results of thalassemia awareness in Romania with a positive impact on the patient’s life, registering the following results:

2000: A website in Romanian language regarding thalassemias and addressed to patients was created.

2005: The Association of People with thalassemia major was founded and become member of Thalassemia International Federation

2011: Thalassemia major patients from Romania have access to free treatment and the latest medical oral chelator, Exjade.

The association participated in various meetings addressed to patients and doctors as well, created their own website, held workshops, fought together for an improved quality of life for themselves, for healthy people (possible thalassemia carriers) and for all Romanian children.

Abbreviations: WWOW – World Wide Opportunities for Woman, APTM – Association of People with thalassemia major, CVS-Chorionic Villus Sampling, TIF – Thalassemia International Foundation

## Introduction

When do people start changing their lifestyle? Most of the time, when something bad happens. That is the time when they realize that something has to be changed in order to have a healthy life and sometimes it is too late to be able to live the healthy life they are dreaming of. When your body is not working properly, it acts like an alarm when it goes off. It wakes you up, sometimes it scares you and the first thing that crosses your mind is to save yourself but if you can have a healthy life on a regular basis the alarm will stay on, your body will be in its best shape, and as well your mind and spirit. So what are we waiting for? Let us do something to improve the quality of life NOW!

## Methods

This is what Word Wide Opportunities for Women, Canada set as objective, in 1999 it when started a 6 months “Health Promotion “course having as participants, Canadian residents with worldwide backgrounds. It started as a new experience and ended as a great success. We begun our course trying to understand each other’s cultural background, talking about ourselves, and share our Canadian experience so far. At a later stage, we started to learn about different subjects. One of them was CPR (Cardiopulmonary Resuscitation). At the end of this class, we were ready to help a person in case of emergency by the time the ambulance or any other emergency unit arrived and we were certified to do so. Later on, we started to learn about nutrition and its impact on our health. We were out at different meetings in this regard and we organized a potluck where each of us took part by bringing healthy dishes cooked at home. We all started to share not only our food and experience but also well new ideas in order to develop our group strategy. In the next step, we were trained for a new job - Fitness instructors. Each of us had the opportunity to work with a wonderful fitness trainer, Sheila Schuehlein. She prepared each of us to become a fitness instructor. Later on, we had to volunteer for some time in a place that promoted health through its activities. It was one more thing that program did for us in order to become fully trained health promoters. We had to prepare a one-hour presentation about what we considered an important subject regarding health promotion. I heard wonderful presentations, about healthy food, about environment etc. It was my turn and I sustained in front of my colleagues a presentation about thalassemia. I had heard about it in Romania, were I was born, but none of my Romanian friends heard about it and as well none of my Canadian friends, so, I considered it an interesting subject.

Starting from this point, from this one-hour presentation, my life and the other people’ life changed. Sometimes we only need a small impulse in life to generate a reaction, a way of thinking with a great impact on the other’s life. That was my time. I picked a subject and now I had to gather information about it. In 1999, we did not have the information we have today on the internet. Actually, not many people had a computer at that time. I was lucky to have one and I was able to create a few contacts in order to find out some things about thalassemia. You may ask yourself ...what is she talking about? What is thalassemia? I will start to tell you what it is with this sentence: Thalassemia minor is an inherited blood condition and in this case, you may have it without knowing about its existence. If your spouse has it without knowing as well...your child’s life is in big danger because he can inherit from his/her parents, what today is called thalassemia major, and this is a disease. From here to your statement: “Well...I do not have...why should I have it...I feel good” it is just one step. To fulfill my health promoter chores I will offer you my presentation about thalassemia.

## Result

Therefore, here is all somebody has to know about THALASSEMIA before they decided to become parents:

**Definition and history**

Dramatic facts in the exciting field of genetics are opening up another front in the battle against birth defects. Thalassemia is a genetic blood disorder that affects a person’s ability to produce hemoglobin. Hemoglobin is the protein in our red blood cells that carry oxygen and nutrients to all parts of the body. Without it, our bodies become weak and unable to thrive. In the past, before the discovery of its worldwide distribution, it was also known as Mediterranean Anemia. It comes from the Greek word “thalasanemia” which means “anemia by the sea”. The thalassemias are among the commonest inherited blood diseases in the world. Over the centuries, they must have accounted for the death of many millions of children. The clinical picture was not recognized as a specific disease until 1925 and then not in the 1 area of the world where the disorder was most common but in the United States by a pediatrician, Thomas Cooley. Following his report, more cases were found and the condition became known as Cooley’s anemia. It is known that thalassemia occurs not only in people from Mediterranean area but also in the Middle East, the Indian subcontinent, South – East Asia and Africa among immigrant populations from these areas. The thalassemias have a distribution concomitant with areas where malaria was common. It is thought that possessing one of the abnormal genes that gives rise to thalassemia gives some protection against malaria. Some doctors believe that this is because the red cells in the body of a thalassemic person are somewhat fragile, so when the malaria parasite gets inside, the red cell breaks down and the parasite stops growing. In normal people (non-thalassemics), the parasite would continue to multiply. Therefore, those people with minor forms of thalassemia appear to have some protection against malaria. In addition, there is much more information available about thalassemia and its effects on the body. This means that medicine can offer more effective treatment than it could in the past. Children born by parents who both carry the trait (thalassemia minor) have a 1 in 4 chance with each pregnancy of inheriting the fatal form of the disorder. Not long ago, children born with Thalassemia seldom survived their first decade of life. Recent medical advances have increased life span. To stay alive, patients must undergo blood transfusions every two to four weeks. Every night patients must insert a needle to receive painful infusions of a special drug for up to twelve hours. Thalassemia has been cured only by bone marrow transplant but this is possible only if there is a donor and even so, it is a risky procedure.

**What are the different kinds of thalassemia?**

There are three clinically types: thalassemia minor, thalassemia intermedia, and thalassemia major. 

• Thalassemia minor, also called thalassemia trait may cause no symptoms, but changes in the blood do occur. A person with thalassemia minor generally has no health problems except for a possible mild anemia, which cannot be corrected with iron supplements.

• Thalassemia intermedia, also called mild Cooley’s anemia is an intermediate form of the disease. Thalassemia intermedia is a clinical condition that varies and must be constantly evaluated by the hematologist. 

• Thalassemia major, also called Mediterranean anemia or Cooley’s anemia, named after the doctor who first described it in 1925 is the most severe form. Thalassemia major is very serious and requires extensive medical care. 

**How do people get thalassemia? **

Thalassemia is an inherited disease, which means it is passed on from parents to their child through their genes. It is not infectious and cannot be “caught” like a cold. It will not develop later in life, nor can a child outgrow it. Both parents must have thalassemia trait in order to pass the disease on to their child, but it only takes one parent to pass trait on to his/her child. Thalassemia trait will never develop into disease. Thalassemia trait can be passed on for many generations without being detected before a child is born with disease. The probabilities to get thalassemia exist for each child independently of what happened with prior children the couple may have had. In other words, each new child has a one-in-four chance of having severe Thalassemia. The inheritance of Thalassemia genes is purely a matter of chance and cannot be altered. 

**Fig. 1:**
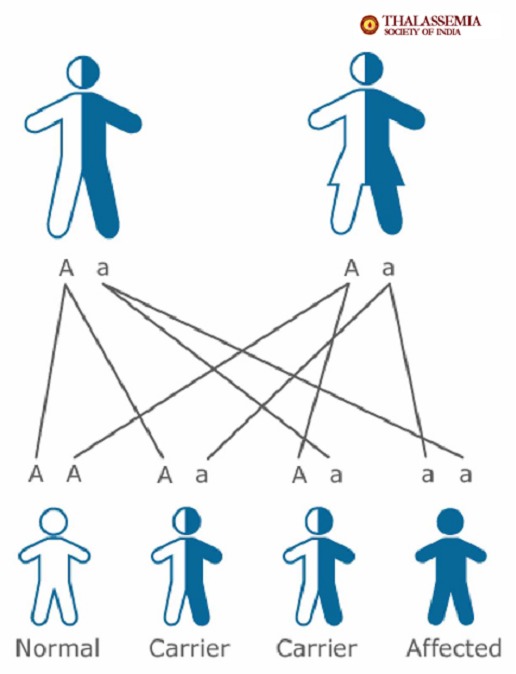
Source: http://thalassemia-india.com/

**Situation no. 1** The main problem that should create the awareness is represented by the result of a marriage when both parents have thalassemia minor (they are called carriers) and do not know about it. As mentioned earlier, thalassemia minor is a health condition but not a disease, you can live with it without knowing about its existence, and, that is why it is so important to know if we are carriers or not because the child of two thalassemia minor patients has one chance out of four to inherit thalassemia major, two chances out of four to inherit thalassemia minor and one chance out of four to be a healthy child. The inheritance possibilities one child has at birth when both parents are carriers are presented in Fig. 1.

**Situation no. 2**

If a thalassemic married a normal, all the children will be healthy carriers. They must inherit a thalassemia gene from their thalassemic parent, but they must also inherit a normal gene from the normal parent, so none of them can possibly have thalassemia major.

**Situation no. 3**

If a thalassemic marries a thalassemia carrier, in each pregnancy there is a 50% chance that the child will be thalassemic, and a 50% chance that it will be a healthy carrier. 

**Situation no. 4**

If one thalassemic marries another, all their children will be thalassemics. 

**How long can people with thalassemia major live? **

These days most thalassemics grow up to become adults, and earn their own living. Most of them find a partner and get married. Now, a number of thalassemia major patients have their own children. The disorder and its influence are changing almost from day to day, because of advances in treatment. Thalassemic patients are now living longer. Today it is reasonable to think that people with thalassemia major, who have been treated from the beginning, may well, live as long as people without thalassemia. Only time will tell. 

**Is there a test for thalassemia? **

Yes. Blood tests and family genetic studies can show whether an individual has thalassemia or is a carrier. In addition, parental testing using chorionic villus sampling (CVS) or amniocentesis can detect thalassemia in the fetus. Early diagnosis is important so that treatment can prevent as many complications as possible. 

**Fig. 2:**
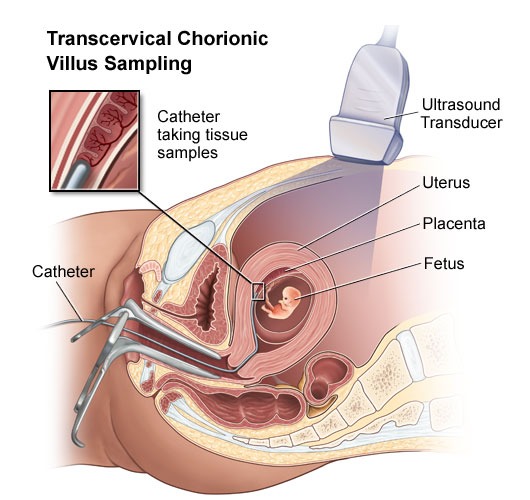
Source: http://www.jeffersonhospital.org/

**• CVS-Chorionic Villus Sample**

CVS allows us to take a small sample of tissue, the chorionic villi, from the developing placenta. CVS is one of the two tests available to check the chromosomes (the structures that contain genes) of the baby. This tissue may also be used for biochemical or direct genetic testing. CVS is performed at 11-12 weeks in the pregnancy and is a day patient procedure. An ultrasound will be performed first to cheek how far pregnant you are, and to show the position of the baby and the developing placenta. As the baby is small and not fully developed, very few physical problems can be detected at this stage by ultrasound scan. The mother's bladder should be full but it does not have to be uncomfortable. 

**CVS diagram **

Chorionic villus can be obtained by inserting a needle through the mother's abdomen (which has been cleaned with antiseptic and numbed with anesthetic) under ultrasound guidance into the developing placenta. A small sample of chorionic villi is withdrawn. Some women experience discomfort during and after this procedure and it is advisable to rest. The chorionic villus sample is sent to the laboratory for testing. A chromosome result will be available in one to two weeks. A biochemical or direct genetic (DNA) test may take longer. CVS carries a small risk of causing a miscarriage. The average risk is 1 chance in 100 to 1 in 200. 

**• Amniocentesis **

Amniocentesis is the removal of a small amount of amniotic fluid from the sac around the baby. This fluid contains cells that come from the baby and the placenta. This test is usually performed at 16 weeks in the pregnancy. Amnio diagram - After cleaning the skin with antiseptic, a fine needle is inserted under ultrasound guidance through the mother's abdomen into a pool of amniotic fluid. A small amount of fluid is withdrawn and the needle removed. Most women say amniocentesis is not painful - however some women feel discomfort. After the procedure, it is advisable to rest for the remainder of the day. You may like to have a family member or friend with you on the day of the test. The amniotic fluid is sent to the laboratory for testing. A chromosome result will take 2-3 weeks. A biochemical or direct genetic (DNA) test may take longer. Amniocentesis carries a small risk of causing a miscarriage. The rate of miscarriage is one in 200 to 1 in 400 for amniocentesis

**What will happen after the test?**

There is a risk of naturally occurring miscarriage in all pregnancies. The risk is highest in early pregnancy and increases with the age of the mother. Most people have normal results after the test. However, if the test shows that the baby has a problem, you will have the opportunity to discuss the results with your doctor, genetic counselor or geneticist. 

**What stages does a woman with thalassemia go through before, during and after pregnancy?**

For a woman with thalassemia major to have children she must have a normal sexual development. Many young women with thalassemia are not having their periods, or whose periods have started and then stopped. If they are not physically fit, a pregnancy could be risky for them and the baby. An expected mother should be fit, meaning they must use their pump regularly and her heart and liver should not have been damaged by iron overload. However, even if they are not perfectly fit there is a chance that they could have a normal pregnancy. Women are advised to stop Desferal (Desferal is a medication that removes this excess of iron from the body) when they are trying to become pregnant, or as soon as they are pregnant. There is no evidence that Desferal can harm the fetus, but in overall, it is a good idea for any pregnant woman to stop taking drugs during pregnancy. Mothers who breastfeed, can start taking Desferal again as soon as the baby is born. Desferal does not pass from the mother’s body onto milk and so cannot harm the baby. Before a woman with thalassemia decides to have a baby, she must consider the long-term future, her own health, survival or whether she will have support from her family. 

**Fig. 3:**
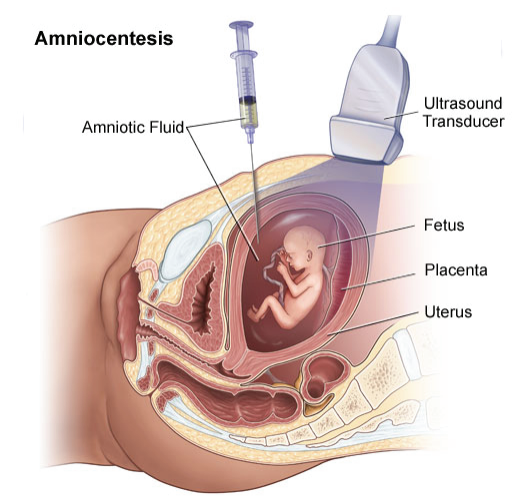
Source: http://www.jeffersonhospital.org/

**What is the major treatment now? **

The treatment for thalassemia major is regular blood transfusions, usually every three or four weeks. Most children, who have these transfusions, usually every three or four weeks, grow normally and live quite happily into their early twenties. However, to live longer, they need other treatments as well. After each blood transfusion, the red cells in the new blood are broken down slowly over the next four months. The iron from the red blood cells stays in the body. If this damage is not prevented most people with thalassemia major die before they reach twenty years old. In 1999, the only way to remove the extra iron from the body was to give injections of a drug called Desferal (Desferrioxamine). Desferal is injected under the skin. The injections are given using a portable battery operated pump. The pump has controls that allow you to set the time for emptying the syringe, and an alarm that warns you if the syringe is stuck, or when the drug has all been injected. The pump is used 5-7 nights of every week. Usually the parents are responsible for this until the child is able to take over. Desferal picks up the iron and carries it out in the urine. In 2011, Exjade [**1**], the first oral chelator [**2,3**], was replacing Desferal in most thalassemia major cases. 

**Is the treatment effective? **

This treatment is very successful and most children treated with blood transfusions and Desferral or Exjade can now lead fairly normal healthy lives. However, the treatment is unpleasant and often upsetting, it also interferes with their desire for an active social life and sometimes medication is neglected. 

**Do thalassemia person need to be on a special diet? **

Thalassemia major patients should try to keep away from foods high in iron such as red meat, liver, kidney, green leafy vegetables such as spinach, some breakfast cereals, wholemeal breads and alcohol. Although this is recommended, patients do not have to stick with this diet. 

**What research in thalassemia is taking place? **

Treatment today is more advanced then what it was. Scientists are working on better ways to remove excess iron from the body in order to prevent or delay iron overload. They are developing and testing the effectiveness of oral iron-chelating drugs, which could greatly simplify treatment of this disease and they are seeking to develop an effective form of gene therapy that may someday offer a cure for thalassemia. Gene therapy may involve inserting a normal beta globin gene (the gene that is abnormal in this disease) into the patient’s stem cells the immature bone marrow cells that are the precursors of all other cells in the blood. Another form of gene therapy may involve using drugs or other methods to reactivate the patient’s genes responsible with production of fetal hemoglobin. All humans produce a fetal form of hemoglobin before birth; after birth, natural genetic switches “turn off” production of fetal hemoglobin and “turn on” production of adult hemoglobin. Scientists are seeking ways to activate these genetic switches so that they can make the blood cells of patients with thalassemia produce more fetal hemoglobin to compensate for their deficiency of adult hemoglobin. Initial studies of rare individuals with genetic traits that allow them to produce only fetal hemoglobin show that they are generally healthy, demonstrating that fetal hemoglobin can be a fine substitute for adult hemoglobin. In addition, improved bone marrow transplantation methods may lead to wider use of the technique as a treatment for thalassemia. Bone marrow transplants have cured some cases of thalassemia but they are not widely used. 

**Can thalassemia be prevented? **

The disease cannot be prevented at this time, but a program of health education, testing for the trait, genetic counseling, and prenatal diagnosis can provide families with full medical information to help them have healthy children. People who think they may have or carry thalassemia can go to a genetic services center or clinic for the latest information and for testing. Individuals can be tested to find out if they are carriers. There is one blood test that shows if a person has thalassemia minor and this is called: “hemoglobin electrophoresis” [**4**]. Genetic counselors then can help them make plans about future families. 

**Why does a thalassemia patient need genetic counseling?**

Genetic counseling is a communication process of providing information and support to families, couples, or individuals that are in some way impacted by an inherited disease such as thalassemia. A genetic counselor often acts as a liaison for communicating complicated medical and genetic information. A non-directive approach can help counselors integrate this information into their own system of beliefs and values. In this way, counselors can make informed personal decisions about genetic testing, health care, and reproduction that make sense for them and their families. 

**Iron deficiency or thalassemia?**

Thalassemia and iron metabolism are closely linked. Iron deficiency and mild forms of thalassemia (i.e. thalassemia trait) are often confused. Both are associated with mild to moderate anemia and microcytosis (smaller red cells). At the other end of the spectrum, severe forms of thalassemia frequently produce iron overload. Excess iron accumulates due to enhanced iron absorption produced by thalassemia, repeated blood transfusions or both. A number of questions are frequently asked regarding thalassemia and iron. 

**Should a person with thalassemia trait avoid iron, such as iron-fortified vitamins? **

Iron replacement tablets or iron-supplemented vitamins should be taken only if prescribed by a physician to treat actual iron deficiency or to prevent iron deficiency in high-risk circumstances (i.e. pregnancy). People with thalassemia trait (thalassemia minor) are not having a greater risk of complications from iron in the diet than anyone else in the general population. There are instances, however, in which coincident conditions can increase the risk of iron overload. 

**Can the anemia produced by thalassemia be corrected or improved by taking more iron? **

In the absence of concomitant iron deficiency, iron supplementation will neither correct nor improve anemia due to thalassemia. For people with both iron deficiency and thalassemia, iron replacement will decrease the severity of the anemia, until the iron deficiency is corrected. The blood count will level off and no further improvement will occur. 

**What can parents do?**

Consult your doctor when you see signs and symptoms suggestive of anemia (thalassemia). Inform your doctor about your family history of thalassemia. Treatment should not begin until your doctor will examine the results of laboratory tests. Make sure your child maintains a well balanced diet. Have the electrophoresis of hemoglobin test done.

**Discussion**

In 2011, there were two drugs which were approved to treat iron overload in Romania – Desferal and Exjade.

• Desferal (deferoxamine) is an effective medication for removing iron from the body. It must be administered slowly by needle, in the vein or under the skin, for eight to 12 hours per day, five to seven days a week. Desferal has significantly changed the prognosis of patients with thalassemia major, but many of the patients find the infusions of Desferal difficult or painful and are reluctant to comply with their doctor’s orders. These patients are not free of risk and may die prematurely due to iron overload.

• Exjade (deferasirox) is the first oral iron chelator available in Romania. A patient dissolves an effective, well-tolerated pill in water and drinks once a day. Exjade improves the patients’ quality of life and may lead to improved patient compliance with treatment.

After I had presented this material to my colleagues in Canada, I started to build a website and translated all the presentation in Romanian language. The result was rewarding. It was the first website in Romanian language regarding thalassemia addressed to patients. Therefore, many parents contacted me to ask me more questions, others to thank me and I would say now that this was the best project in my entire life. I supported APTM throughout its foundation process and I created a permanent link between Thalassemia International Federation (TIF), Cyprus and APTM. That way I was able to promote health and improve the quality of life for some Romanian families...and who knows...maybe for families from other country as well. My work did not stop here. There is still so much to do... From now on, I invite everybody to join us and continue to promote health next to APTM and myself by:

• Creating all the needed support for the Romanian Ministry of Health in order to implement a screening program for thalassemia and other hemoglobinopathies [**5**] in parallel with an awareness program.

• promoting health and awareness in any possible way: Updated website (It is our hope that by providing electronic education about the disease, we can raise awareness, encourage people to get tested for trait, and spread knowledge about the latest news in this regards), workshops for medical stuff and patients, presentations in high schools and universities creating the awareness that any of us can be a thalassemia carrier and it is time to become more responsible for our children’s life and take the electrophoresis of hemoglobin test before marriage. 

**Disclaimer**

This research and its content are provided here as information only. If you have any questions about your health, please contact your health care provider. The author presents this data as is, without any warranty of any kind, express or implied and is not liable for mistakes, errors, or omissions.
